# Correlation study of CAR, PLR, NLR with the prognosis of cardiogenic cerebral embolism patients

**DOI:** 10.3389/ebm.2025.10517

**Published:** 2025-06-17

**Authors:** Xiaojing Du, Xiaohui Li, Sheng Yue, Yuzhen Sun, Mengzhen Zhao, Lingshan Zhou, Xingwei Wang, Yapan Yang

**Affiliations:** ^1^ Department of Comprehensive Intensive Care Unit, Central China Fuwai Hospital, Fuwai Central China Cardiovascular Hospital, Central China Fuwai Hospital of Zhengzhou University, Heart Center of Henan Provincial People’s Hospital, Zhengzhou, Henan, China; ^2^ Department of Cardiology, Central China Fuwai Hospital, Fuwai Central China Cardiovascular Hospital, Central China Fuwai Hospital of Zhengzhou University, Heart Center of Henan Provincial People’s Hospital, Zhengzhou, Henan, China

**Keywords:** cardiogenic cerebral embolism, prognosis, CAR, PLR, NLR

## Abstract

This study explored the association between inflammatory biomarkers—C-reactive protein to albumin ratio (CAR), platelet to lymphocyte ratio (PLR), and neutrophil to lymphocyte ratio (NLR)—and the prognosis of patients with cardiogenic cerebral embolism (CCE). We retrospectively analyzed data from 80 CCE patients diagnosed between June 2020 and June 2024, categorizing them into favorable and unfavorable prognosis groups based on outcomes such as death, recurrence, and disability. The CAR, PLR, and NLR values were calculated from routine blood tests, and statistical analyses, including Spearman correlation, multivariate logistic regression, and ROC curve analysis, were performed to examine their prognostic significance. Results showed that the unfavorable prognosis group had significantly higher CAR, PLR, and NLR values compared to the favorable group (P < 0.05). Spearman correlation analysis revealed positive associations between these biomarkers and prognosis (r = 0.319 for CAR, 0.238 for PLR, 0.251 for NLR, all P < 0.05). Multivariate analysis identified CAR and NLR as independent risk factors for unfavorable prognosis (OR = 1.034 for CAR, OR = 3.887 for NLR). ROC analysis determined optimal cutoff values for CAR (>0.74), PLR (>160.00), and NLR (>3.53) to predict unfavorable prognosis with AUCs of 0.796, 0.694, and 0.705, respectively. The combined biomarker test yielded an AUC of 0.899. Kaplan-Meier survival analysis indicated significantly lower survival rates for patients with higher levels of CAR, PLR, and NLR (P < 0.05). In conclusion, elevated CAR, PLR, and NLR are reliable indicators of a poor prognosis in CCE patients.

## Graphical Abstract

**Figure:**
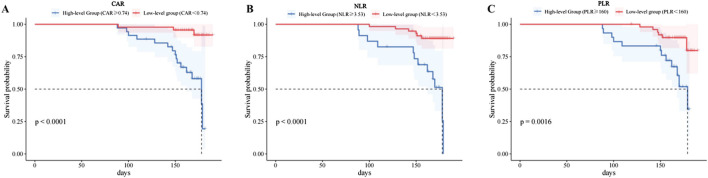
Cardiac cerebral embolism (CCE) is a serious cerebrovascular disease characterized by the detachment of blood clots from the heart. This study collected clinical data from 80 CCE patients for analysis and found that 18 cases experienced adverse prognostic events (including death, recurrence, or severe disability) within 6 months of follow-up, with an incidence rate of 22.5%. Through in-depth analysis, it was found that CAR, PLR, and NLR are closely related to the prognosis of CCE patients. Therefore, this study suggests that CAR, PLR, and NLR, as easily obtainable biomarkers of inflammatory response in routine clinical practice, combined detection can help identify high-risk CCE patients early and provide important basis for clinical decision-making.

## Impact statement

In recent years, inflammatory biomarkers such as the C-reactive protein to albumin ratio (CAR), platelet to lymphocyte ratio (PLR), and neutrophil to lymphocyte ratio (NLR) have been utilized in the prognostic assessment of various diseases. This study aimed to examine the correlation between CAR, PLR, and NLR, and the prognosis of patients with cardiogenic cerebral embolism (CCE), as well as to evaluate their potential as prognostic predictors.

## Introduction

Cardiac cerebral embolism (CCE) is a significant cerebrovascular condition characterized by the dislodgment of thrombus from the heart. This thrombus can enter the brain via the bloodstream, leading to the obstruction of cerebral blood vessels, which subsequently results in neurological dysfunction, including symptoms such as limb weakness and slurred speech. In severe cases, this condition may lead to permanent neurological impairment [1]. Globally, CCE account for approximately 15%–30% of ischemic strokes and are typically associated with higher morbidity, disability, and mortality rates, posing a significant threat to the lives and health of patients [2]. Consequently, the early identification of high-risk factors for CCE and timely intervention hold critical clinical importance for enhancing patient prognosis.

Brain natriuretic peptide (BNP) and D-dimer are widely utilized biomarkers for diagnosing and monitoring treatment responses in patients with CCE. These biomarkers have been endorsed by authoritative organizations, including the European Society of Cardiology and the American Society of Hematology [3, 4]. However, the high costs associated with BNP and D-dimer testing, coupled with limited availability in some primary medical institutions, underscore the necessity of identifying more economical and straightforward predictive indicators. In recent years, an increasing number of studies have focused on the potential role of inflammatory markers in CCE. Specifically, the C-reactive protein to albumin ratio (CAR), platelet to lymphocyte ratio (PLR), and neutrophil to lymphocyte ratio (NLR) are gaining recognition as promising new biomarkers.

The CAR serves as a comprehensive indicator of both inflammation and nutritional status. An elevation in CAR reflects the body’s inflammatory response and malnutrition, and is associated with poor prognoses in various diseases, including cardiovascular disorders and tumors [5, 6]. In cases of CCE, elevated CAR may indicate more severe cerebrovascular injury and a heightened inflammatory response, both of which are closely linked to poor outcomes. The platelet-to-lymphocyte ratio (PLR) and neutrophil-to-lymphocyte ratio (NLR) are additional indicators that reflect the body’s inflammatory and immune status; their variations may also be significantly related to the occurrence and progression of CCE. An increase in PLR suggests that the body is experiencing inflammation [7], while an increase in NLR indicates an enhanced inflammatory response mediated by neutrophils, coupled with a diminished immune response mediated by lymphocytes [8], This state of immune imbalance may facilitate the formation and shedding of thrombi, thereby elevating the risk of CCE. Therefore, the purpose of this study is to explore the correlation between the levels of the three inflammatory markers, CAR, PLR, and NLR, and the prognosis in patients with CCE. It is hoped that this research can provide new reference criteria for early warning, condition assessment, and prognostic judgment of CCE.

## Materials and methods

### Study method and object

**FIGURE 1:**
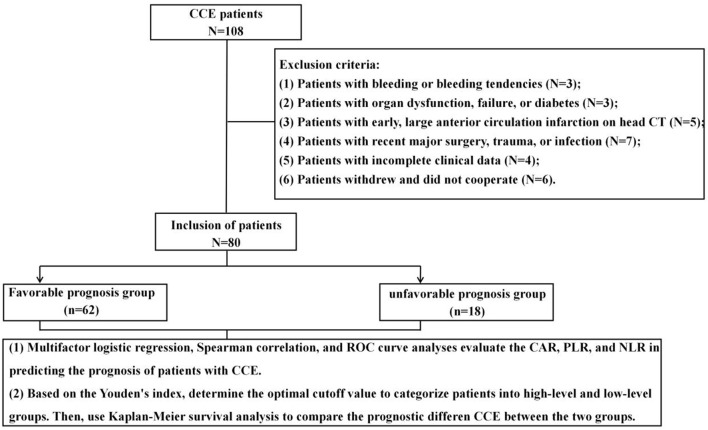
Research technology roadmap.

A total of 108 CCE patients treated at our hospital from June 2020 to June 2024 were considered for this study, from which 80 patients were selected as research subjects based on predefined inclusion and exclusion criteria. The research flow chart is presented in Figure 1. Subsequently, patients were categorized into two groups based on the occurrence of adverse prognostic events (such as death, recurrence, or severe disability) during hospitalization and within a 6-month follow-up period post-discharge: a favorable prognosis group and a unfavorable prognosis group. Among them, severe disability is defined as; Major body organ defects, obvious organ deformities, moderate body organ dysfunction, serious complications, etc. The favorable prognosis group consisted of patients who did not experience any of the aforementioned adverse events during hospitalization or within the 6-month follow-up period; conversely, the unfavorable prognosis group included patients who experienced any adverse prognostic events during this timeframe. All enrolled patients provided informed consent, and this study received approval from Fuwai Central China Cardiovascular Hospital Ethics Committee. (No. 2020-05).

Inclusion criteria: (1) The patient meets the diagnostic criteria for CCE [9]; (2) The patient is diagnosed with CCE through examinations such as head CT, magnetic resonance imaging (MRI), echocardiography, etc; (3) The patient’s baseline National Institutes of Health Stroke Scale (NIHSS) score is ≥6 points; (4) Patients must have complete clinical data and follow-up data.

Exclusion criteria: (1) Patients with active bleeding or known bleeding tendencies; (2) Patients with significant organ dysfunction or failure, or severe diabetes; (3) Patients whose head CT indicates early and extensive infarction in the anterior circulation, specifically those exceeding one-third of the middle cerebral artery blood supply area; (4) Patients with a history of major surgery, trauma, or infection within the past 2 months; (5) Patients who refuse to participate in this study or who are unable to complete follow-up assessments.

### Methods

The general clinical data of all patients were retrospectively collected, encompassing age, gender, height, history of hypertension, diabetes, hyperlipidemia, smoking history, drinking history, the presence of congestive heart failure (CHF), and the NIHSS score recorded upon admission. Additionally, 5 mL of fasting venous blood was collected from each patient within 24 h of admission, which was then centrifuged at 4°C at 3,000 rpm for 30 min to obtain serum samples. These samples were subsequently sent to our hospital’s laboratory department for routine blood count and blood cell calculation. All patients received standardized treatments, including intravenous thrombolysis, anticoagulation, anti-platelet aggregation, lipid regulation and plaque stabilization, as well as management of blood pressure and blood sugar levels, neuronutrition, and symptomatic supportive care. Clinical outcomes were assessed through telephone follow-up or outpatient review within 3 months post-discharge, focusing on adverse prognostic events such as death, recurrence, or severe disability.

#### Routine blood test

Utilizing an automatic biochemical analyzer (model GS480Plus, manufactured by Shenzhen Jinrui Biotechnologies Co., Ltd., registration number 20162220670), we analyzed the levels of uric acid (UA), homocysteine (HCY), creatinine (Cr), triglyceride (TG), low-density lipoprotein (LDL-C), and high-density lipoprotein (HDL-C). Furthermore, the concentrations of serum albumin (ALB) and C-reactive protein (CRP) were assessed via immunoturbidimetry. Subsequently, the CAR was computed based on these measurements.

#### Blood cell count

Using an automatic blood cell analyzer (manufacturer: Shenzhen Pukang Electronics Co., Ltd.; model: PE-7000; registration number: 20182220948), we determined the counts of neutrophils, lymphocytes, and platelets. Additionally, we calculated the NLR and the PLR based on these counts.

### Statistical treatment

Data analysis was performed using SPSS 26.0 software. Normality tests were conducted on continuous variables. Normally distributed continuous data were presented as mean ± standard deviation, and t-tests were used for intergroup comparisons. Categorical data were expressed as counts and percentages, and χ^2^ tests were applied for intergroup comparisons. Multivariate logistic regression analysis was utilized to identify factors influencing the prognosis of CCE patients, with significant variables serving as independent variables and patient prognosis as the dependent variable, to evaluate the predictive power of these factors for prognosis. Moreover, Spearman’s rank correlation analysis was conducted to assess the correlation between each variable and patient prognosis. Receiver operating characteristic (ROC) curves were generated, and the area under the curve (AUC) and Youden’s index were calculated to further validate the predictive performance of CAR, PLR, and NLR, both individually and in combination, for the prognosis of CCE patients. Optimal cut-off values were determined based on Youden’s index, and patients were categorized into high-level and low-level groups. Kaplan-Meier survival analysis was subsequently employed to compare the prognostic differences between these groups. A *P*-value of less than 0.05 was deemed to indicate statistical significance.

## Results

### Patient characteristics

A total of 80 CCE patients were included in the study, of whom 18 experienced adverse prognostic events (including death, recurrence, or severe disability) within 6 months of follow-up and were classified as the unfavorable prognosis group. The remaining 62 patients were classified as the favorable prognosis group. The baseline characteristics of the two patient groups are detailed in Table 1. In the unfavorable prognosis group, the average age of patients and the NIHSS score were higher, and the proportion of patients with hypertension and congestive heart failure was also greater than that in the favorable prognosis group, with these differences reaching statistical significance (*P* > 0.05).

**TABLE 1:** Comparison of baseline characteristics between the two patient groups.

Characteristics	Type	Favorable prognosis group (n = 18)	Unfavorable prognosis group (n = 62)	*P*
Age		52.00 ± 8.20	45.76 ± 8.29	0.006
Sex	Man	11 (61.11)	48 (77.42)	0.166
	Woman	7 (38.89)	14 (22.58)	
Hypertension	Yes	8 (44.44)	10 (16.13)	0.011
	Deny	10 (55.56)	52 (83.87)	
Diabetes mellitus	Yes	4 (22.22)	7 (11.29)	0.236
	Deny	14 (77.78)	55 (88.71)	
Hyperlipemia	Yes	5 (27.78)	15 (24.19)	0.757
	Deny	13 (72.22)	47 (75.81)	
History of smoking	Yes	6 (33.33)	16 (25.81)	0.529
	Deny	12 (66.67)	46 (74.19)	
History of drinking	Yes	5 (27.78)	25 (40.32)	0.333
	Deny	13 (72.22)	37 (59.68)	
CHF	Yes	8 (44.44)	9 (14.52)	0.006
	Deny	10 (55.56)	53 (85.48)	
NIHSS score at admission		15.83 ± 3.82	13.42 ± 3.50	0.014

Note: Measurement data in normal distribution in x ± s, t-test for comparison between groups; count data in n and% for χ^2^checkout.CHF, Congestive heart failure; NIHSS, national institutes of health stroke scale.

### Laboratory indicators

Further analysis of the laboratory indicators for the two patient groups revealed that the values of CAR, PLR, and NLR in the unfavorable prognosis group were significantly higher than those in the favorable prognosis group, with the differences being statistically significant (*P* < 0.05), as shown in Table 2.

**TABLE 2:** Comparison of the experimental indicators between the two patient groups.

Characteristics	Favorable prognosis group (n = 18)	Unfavorable prognosis group (n = 62)	*P*
Neutrophil count (10^9^/L)	7.18 ± 0.97	6.74 ± 0.92	0.077
Lymphocyte count (10^9^/L)	1.41 ± 0.32	1.68 ± 0.56	0.050
Platelet count (10^9^/L)	227.94 ± 16.54	216.84 ± 21.98	0.051
C-reactive protein (mg/L)	22.32 ± 6.76	25.95 ± 6.87	0.051
ALB (g/L)	38.07 ± 7.62	33.88 ± 8.34	0.059
CAR	0.80 ± 0.26	0.60 ± 0.21	0.004
PLR	170.02 ± 38.00	143.21 ± 48.32	0.034
NLR	4.62 (3.78, 5.84)	3.09 (2.38, 5.23)	0.008
UA (μmol/L)	305.34 ± 24.09	314.35 ± 26.31	0.197
HCY(μmol/L)	13.58 ± 3.15	13.08 ± 2.98	0.534
Cr (μmol/L)	65.66 ± 4.81	66.42 ± 5.04	0.570
TG (mmol/L)	1.48 ± 0.38	1.48 ± 0.38	0.960
LDL (mmol/L)	2.88 ± 0.76	2.67 ± 0.60	0.234
HDL (mmol/L)	1.10 ± 0.25	1.17 ± 0.30	0.347

Note: Measurement data conform to normal distribution are expressed as‾x ± s, t-test for comparison between groups; measurement data are not normally distributed as [M (P_25_, P_75_)], non-parametric rank sum test; count data are presented as n and%, χ^2^checkout.CAR, C-reactive protein to albumin ratio; NLR, neutrophil-to-lymphocyte ratio; PLR, platelet-to-lymphocyte ratio; UA, uric acid; HCY, Homocysteine; Cr, Creatinine; TG, Triglyceride; LDL, low-density lipoprotein; HDL, high-density lipoprotein.

### Correlation analysis of adverse prognosis in patients with CCE

Pearson’s correlation analysis revealed that age, NIHSS score, hypertension, congestive heart failure, CAR, PLR, and NLR were significantly associated with unfavorable prognosis in patients. Specifically, age, NIHSS score, and increases in CAR, PLR, and NLR demonstrated strong correlations with unfavorable prognosis, with correlation coefficients of r = 0.304, 0.274, 0.319, 0.238, and 0.251, respectively (*P* < 0.05). Additionally, patients with both hypertension and congestive heart failure exhibited a significantly elevated risk of adverse prognosis, with correlation coefficients of r = 0.283 and 0.306 (*P* < 0.05), as shown in Table 3.

**TABLE 3:** Association with unfavorable prognosis in patients with CES.

Project	Statistical value	Age	NIHSS grade	Hypertension	CHF	CAR	PLR	NLR
prognosis	*r*	0.304	0.274	0.283	0.306	0.319	0.238	0.251
	*P*	0.006	0.014	0.011	0.006	0.004	0.034	0.024

Note: NIHSS, national institutes of health stroke scale; CHF, Congestive heart failure; CAR, C-reactive protein to albumin ratio; NLR, neutrophil-to-lymphocyte ratio; PLR, platelet-to-lymphocyte ratio.

### Multivariate logistic regression analysis of factors affecting adverse prognosis in patients with CCE

In order to pinpoint the independent risk factors for unfavorable outcomes in patients with CCE, a multivariate logistic regression analysis was performed. Variables that exhibited significant differences in the univariate analysis, including age, NIHSS score, hypertension, congestive heart failure, CAR, PLR, and NLR, were incorporated into the multivariate logistic regression model. The analysis revealed that age (OR = 1.095, 95% CI: 1.022–1.173, *P* = 0.010), NIHSS score (OR = 1.321, 95% CI: 1.013–1.725, *P* = 0.040), CAR (OR = 1.034, 95% CI: 1.002–1.067, *P* = 0.037), and NLR (OR = 3.887, 95% CI: 1.163–12.993, *P* = 0.027), are independent predictors of adverse outcomes in CCE patients. For more detailed information, refer to Table 4.

**TABLE 4:** Multifactor logistic analysis of adverse prognosis in patients with CCE.

Variable	*β*	*SE*	Waldχ^2^	*P*	OR (95%CI)
Age	0.091	0.035	6.640	0.010	1.095 (1.022–1.173)
NIHSS score at admission	0.279	0.136	4.209	0.040	1.321 (1.013–1.725)
Hypertension	1.455	0.955	2.321	0.128	4.283 (0.659–27.827)
CHF	0.489	1.028	0.226	0.634	1.631 (0.218–12.221)
CAR	0.033	0.016	4.338	0.037	1.034 (1.002–1.067)
PLR	0.016	0.009	2.959	0.085	1.016 (0.998–1.034)
NLR	1.358	0.616	4.861	0.027	3.887 (1.163–12.993)

Note: NIHSS, national institutes of health stroke scale; CHF, Congestive heart failure; CAR, C-reactive protein to albumin ratio; NLR, neutrophil-to-lymphocyte ratio; PLR, platelet-to-lymphocyte ratio.

### ROC curve analysis of factors affecting unfavorable prognosis in patients with CCE

**FIGURE 2:**
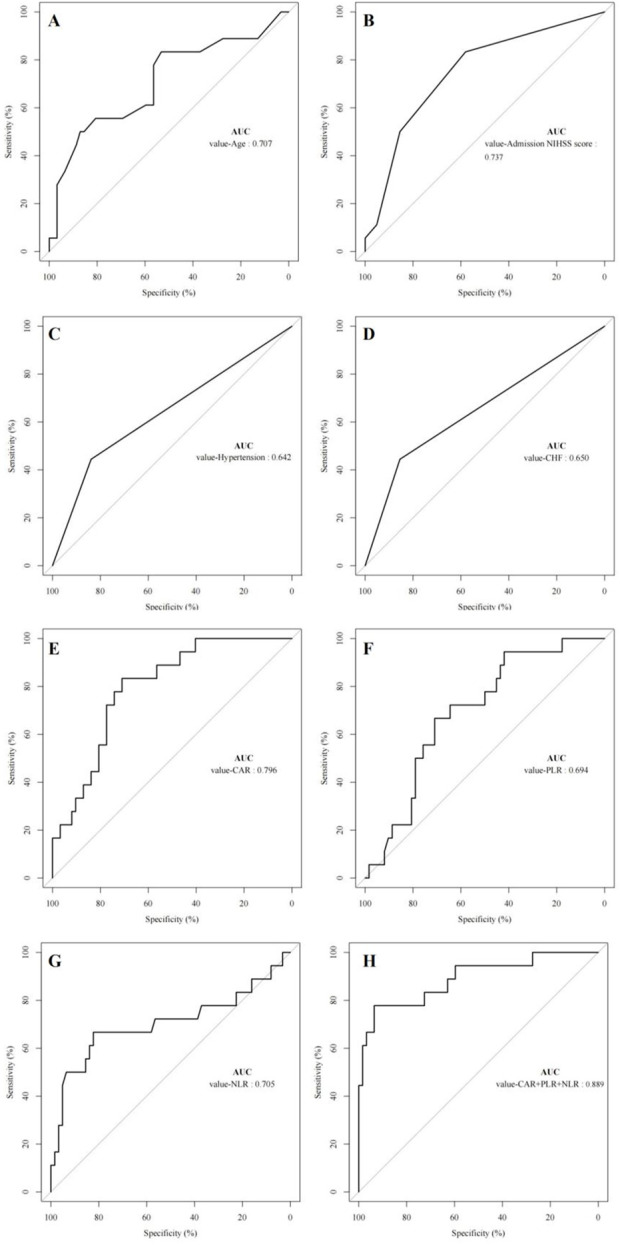
ROC curve analysis affecting the prognosis of CCE patients. Note: NIHSS, national institutes of health stroke scale; CHF, Congestive heart failure; CAR, C-reactive protein to albumin ratio; NLR, neutrophil-to-lymphocyte ratio; PLR, platelet-to-lymphocyte ratio. **(A)** The AUC of age, **(B)** The AUC of NIHSS, **(C)** The AUC of hypertension, **(D)** The AUC of CHF, **(E)** The AUC of CAR, **(F)** The AUC of PLR, **(G)** The AUC of NLR, **(H)** The AUC of CAR+PLR+NLR.

To further evaluate the predictive power of the aforementioned variables for the prognosis of CCE patients, a ROC curve analysis was conducted. The results indicated that age, NIHSS score at admission, CAR, PLR, and NLR possess significant predictive capabilities for adverse outcomes in CCE patients, as detailed in Table 5 (*P* < 0.05). Specifically, the AUC values for age, NIHSS score at admission, CAR, PLR, and NLR were 0.707, 0.737, 0.796, 0.694, and 0.705, respectively, each demonstrating notable predictive value, as illustrated in Figure 2. Notably, CAR exhibited the highest AUC, signifying its superior predictive efficacy for adverse outcomes in CCE patients. Further analysis revealed that the combined use of CAR, PLR, and NLR resulted in an elevated AUC value of 0.889 (95% CI: 0.792–0.986, *P* < 0.05), accompanied by relatively high sensitivity and specificity of 93.5% and 77.8%, respectively. This finding underscores that the joint predictive application of CAR, PLR, and NLR can significantly enhance the precision of prognostic predictions for CCE patients.

**TABLE 5:** ROC curve analysis of factors affecting adverse prognosis in patients with CCE.

Variable	AUC	*SE*	*P*	95% CI	Sensitivity (%)	Specificity (%)	Youden’s index
Age	0.707	0.076	0.008	0.559–0.855	50.0	87.1	0.371
NIHSS score at admission	0.737	0.070	0.002	0.599–0.875	88.7	50.0	0.387
hypertension	0.642	0.079	0.069	0.486–0.797	83.9	44.4	0.283
CHF	0.650	0.079	0.054	0.494–0.805	85.5	44.4	0.299
CAR	0.796	0.053	<0.001	0.692–0.899	71.0	83.3	0.543
PLR	0.694	0.064	0.013	0.569–0.819	66.7	71.0	0.377
NLR	0.705	0.085	0.008	0.538–0.871	82.3	66.7	0.490
CAR + PLR + NLR	0.889	0.050	<0.001	0.792–0.986	93.5	77.8	0.713

Note: NIHSS, national institutes of health stroke scale; CHF, Congestive heart failure; CAR, C-reactive protein to albumin ratio; NLR, neutrophil-to-lymphocyte ratio; PLR, platelet-to-lymphocyte ratio.

### Kaplan-Meier survival analysis of CAR, PLR, and NLR levels on the prognosis of CCE patients

**FIGURE 3:**
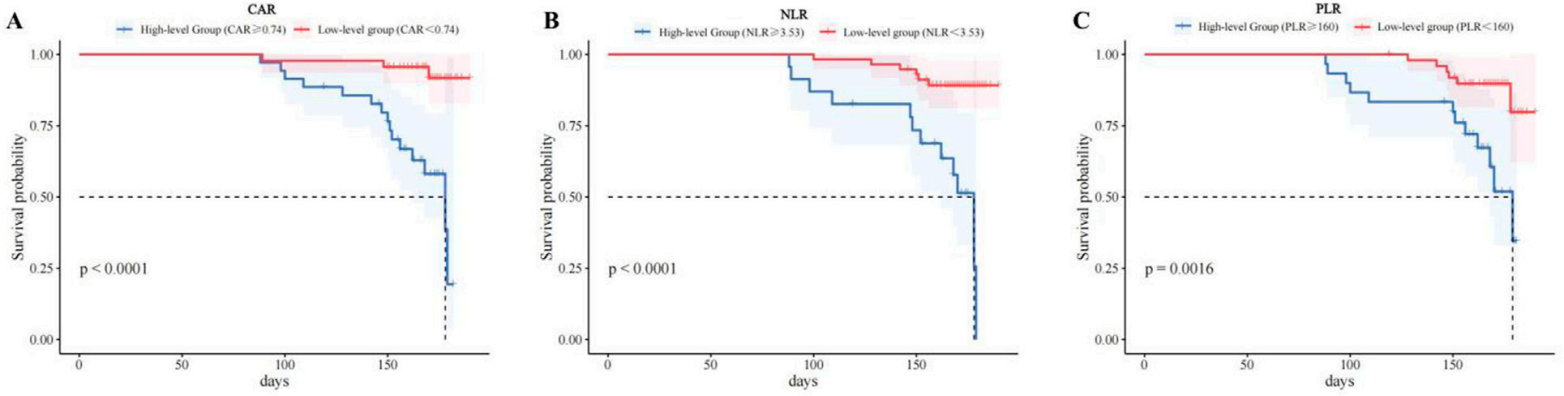
Kaplan-Meier survival curves of CCE patients with different CAR, PLR, and NLR levels. Note: CAR, C-reactive protein to albumin ratio; NLR, neutrophil-to-lymphocyte ratio; PLR, platelet-to-lymphocyte ratio. **(A)** The Kaplan-Meier of CAR, **(B)** The Kaplan-Meier of NLR, **(C)** The Kaplan-Meier of PLR.

According to the Youden index, the optimal cutoff values for CAR, PLR, and NLR are 0.74, 160.00, and 3.53, respectively. The CAR, PLR, and NLR levels were categorized into two groups: high-level and low-level. Kaplan-Meier survival analysis was employed to compare the prognostic differences between these two patient groups. The results indicated that the survival rates of patients in the high-level CAR group (≥0.74), high-level PLR group (≥160.00), and high-level NLR group (≥3.53) were significantly lower than those in the low-level group, with the differences between the two groups being statistically significant (*P* < 0.05), as shown in Figure 3.

## Discussion

CCE is characterized by inadequate cerebral blood perfusion due to cardiogenic factors, including atrial fibrillation, myocardial infarction, and valvular heart disease. These factors can lead to brain tissue damage resulting from ischemia, hypoxia, and, in severe cases, necrosis. The disease is marked by sudden onset and rapid progression, making early diagnosis and intervention essential to minimize disability and mortality rates among patients [10]. Furthermore, inflammation plays a significant role in the onset and progression of CCE. Inflammatory mediators, such as cytokines, chemokines, and adhesion molecules, can damage vascular endothelial cells, thereby promoting thrombosis [11]. The inflammatory response may also activate platelets and the coagulation system, increasing blood coagulability and exacerbating the ischemic and hypoxic conditions of brain tissue [12]. Consequently, in clinical practice, the condition and prognosis of CCE patients can be assessed by monitoring changes in inflammatory indicators. This study focuses on easily accessible and straightforward experimental indicators, such as the CAR, PLR, and NLR, to investigate the correlation between these inflammatory markers and the prognosis of CCE patients.

The results of this study showed that of the 80 patients with CES, 18 experienced adverse outcome events (including death, recurrence or severe disability) within 6 months of follow-up, and the incidence of adverse outcome events was 22.5%. Studies show that the in-hospital mortality rate in patients with CES is 27.3% [13], This data closely aligns with the incidence of adverse prognostic events observed in this study, thereby reinforcing the high-risk nature of CES patients. The clinical data and experimental indicators of patients categorized into unfavorable and favorable prognosis groups were analyzed in depth. The analysis revealed that the mean age, NIHSS score at admission, and the proportion of patients with combined hypertension and congestive heart failure were significantly higher in the unfavorable prognosis group compared to those in the favorable prognosis group. Notably, among patients with acute ischemic stroke (AIS), the mean age in the unfavorable prognosis group was 74.00 years, whereas it was only 66.00 years in the favorable prognosis group [14]. Elderly patients often experience physiological decline and a reduction in metabolic rate, among other factors, which contribute to their relatively weak resistance to diseases and diminished rehabilitation capabilities. Consequently, this increases the likelihood of unfavorable prognoses. Additionally, the NIHS) score upon admission serves as a crucial indicator for assessing patient outcomes. A higher NIHSS score signifies more severe neurological deficits, which are typically associated with poorer prognoses [15]. In addition, patients with hypertension or congestive heart failure experience a significant decline in heart function, which elevates the risk of developing heart disease and associated complications, such as coronary artery disease and diabetes, thereby increasing the likelihood of adverse outcomes. In the experimental indicators, the values of CAR, PLR, and NLR in the unfavorable prognosis group were significantly higher than those in the favorable prognosis group, demonstrating a notable positive correlation with unfavorable prognosis (r = 0.006, 0.004, and 0.034). These findings align with previous studies [16], suggesting that these inflammatory indicators possess substantial predictive value concerning the prognosis of CES.

CAR is the ratio of CRP to ALB, which reflects, to some extent, the balance between the body’s inflammatory response and nutritional status. CRP, an acute-phase response protein, typically exhibits elevated levels during an inflammatory response in the body [17]. While ALB acts as a negative acute phase reactant, its reduced levels may reflect protein loss due to the poor nutritional status of the organism or the presence of an inflammatory response [18]. Consequently, an increase in CAR levels may signify an exacerbation of the inflammatory response and suggest a deterioration in the patient’s nutritional status. Relevant studies suggest that CAR is more effective in indicating inflammatory status than CRP or albumin alone [19]. Yu et al. [20] found that in patients with acute ischemic cerebral infarction (AIS), the CAR is associated with adverse clinical manifestations of AIS, with patients exhibiting high CAR values experiencing higher mortality rates. This finding is consistent with the results of the current study. Furthermore, this study confirmed that CAR is a significant factor influencing the poor prognosis of patients with CCE through multifactorial logistic regression analysis and ROC curve analysis. Specifically, the odds ratio (OR) for CAR was 1.034 (95% CI: 1.002–1.067), and the AUC reached 0.796, indicating that CAR possesses a high predictive value in CCE. Additionally, Kaplan-Meier survival analysis demonstrated that the survival time of patients with a CAR value greater than 0.74 was significantly shorter than that of patients with lower CAR values, further underscoring the importance of CAR in assessing the prognosis of CCE patients.

Similarly, elevated NLR and PLR values are recognized as independent risk factors for poor prognosis in various cardiovascular diseases [21]. PLR, which reflects the ratio of platelets to lymphocytes, serves as a comprehensive indicator of platelet activation and immune status. The activation of platelets plays a crucial role in thrombosis, while lymphocytes are integral to the body’s immune response [22, 23]. An elevated PLR may indicate that the body is experiencing an inflammatory response or is in an environment conducive to thrombosis. In the multivariate logistic regression analysis conducted in this study, the OR for PLR was found to be 1.016 (95% CI: 0.998–1.034), although this did not achieve statistical significance. This finding aligns with the results reported by Vakhshoori et al. [24]. While Zhai et al [25] posited that the platelet-to-lymphocyte ratio (PLR) serves as an independent predictor of in-hospital mortality among patients in the cardiac intensive care unit (CICU). An increase in PLR is significantly associated with higher in-hospital mortality rates, as well as prolonged lengths of stay in both the CICU and the hospital. Subsequent ROC curve analysis yielded an AUC value of 0.694, suggesting that PLR possesses a moderate predictive capability regarding poor prognosis in patients with CCE, albeit slightly inferior to that of the CAR. This discrepancy may be attributed to the intricate pathophysiological mechanisms underlying CCE, including factors such as the nature, size, and location of emboli, as well as the patient’s underlying conditions and immune status. Furthermore, patients with CCE frequently present with multiple comorbidities, such as hypertension and diabetes, which could influence PLR levels and thereby confound its direct association with mortality risk. Consequently, future research should involve larger sample sizes, extended follow-up durations, and more stringent statistical methodologies to further investigate the prognostic value of PLR in CCE. Additionally, integrating other inflammatory markers (e.g., CAR, NLR) and clinical indicators may enhance the accuracy of prognostic assessments.

NLR serves as a biomarker for inflammatory status. Following the occurrence of CCE, neutrophils are rapidly recruited to the ischemic injury site to participate in the inflammatory response, resulting in a significant increase in their numbers. Concurrently, the number of lymphocytes may decrease due to factors such as the stress response, leading to an elevated NLR and an increased risk of poor prognosis [26]. This result is consistent with previous studies [24, 27], which all confirmed the important value of NLR in the prognosis evaluation of cardiovascular diseases. The normal NLR range is typically 1 to 2. Values above 3.0 or below 0.7 may indicate pathology. Previous studies have confirmed the prognostic significance of NLR in cardiovascular disease [28]. Quan et al. [29] included 590 patients with acute ischemic stroke and found that an elevated NLR of 3.872 serves as a predictive indicator for malignant hemorrhagic transformation, poor functional outcomes, and short-term mortality. In our study, a similar finding was observed, with an increased mortality rate among CCE patients when NLR exceeded 3.5. This finding corroborates the reliability of NLR as a prognostic tool for CCE patients. Moreover, our study revealed that the combined detection of CAR, PLR, and NLR may further enhance the predictive accuracy for the prognosis of CCE patients. Although each individual marker has demonstrated certain predictive efficacy, the integration of multiple indicators allows for a comprehensive assessment of the body’s inflammatory response, coagulation status, and immune condition, thereby providing a more holistic evaluation of patient prognosis. Therefore, in future clinical practice, we propose considering the combined detection of CAR, PLR, and NLR as an important means of prognostic assessment for CCE patients. This approach is expected to further improve treatment outcomes and quality of life for these patients.

In summary, the CAR, PLR, and NLR are biomarkers of inflammatory response that can be easily obtained in routine clinical practice. When used in combination, their detection can facilitate the early identification of high-risk patients with CCE and provide a crucial basis for clinical decision-making. Although this study yielded meaningful findings, several limitations must be acknowledged. First, the study was retrospective, conducted at a single center, and involved a small sample size, which may introduce selection bias and reporting bias. Secondly, the follow-up period was relatively short and may not adequately reflect the long-term prognosis of the patients. Finally, due to the limitations of retrospective studies, the influencing factors that can be collected in this study are limited, and there is a lack of analysis on factors such as treatment changes, admission time, or stroke severity beyond NIHSS scores. Therefore, future research should involve larger-scale, multi-center, prospective studies to further validate the prognostic value of these inflammatory markers in CCE. Additionally, it is essential to explore other factors that may influence prognosis in order to provide patients with a more comprehensive and accurate assessment and treatment strategy.

## Data Availability

The original contributions presented in the study are included in the article/supplementary material, further inquiries can be directed to the corresponding author.
